# Editorial: BET proteins in chromatin architecture, transcription and disease

**DOI:** 10.3389/fmolb.2022.977812

**Published:** 2022-07-22

**Authors:** Amit Kumar Singh, Sheetal Uppal, Keiko Ozato, Dinah S. Singer, Ballachanda N. Devaiah

**Affiliations:** ^1^ Experimental Immunology Branch, NCI, NIH, Bethesda, MD, United States; ^2^ Molecular Biology Division, Bhabha Atomic Research Centre, Mumbai, India; ^3^ Homi Bhabha National Institute, Mumbai, India; ^4^ Laboratory of Molecular Growth Regulation, NICHD, NIH, Bethesda, MD, United States

**Keywords:** BET proteins, BRD4, NIPBL, respiratory syncytial virus, BET inhibitors, metabolic diseases, human papilloma viruses, CNS diseases

The bromodomain and extra terminal (BET) protein family has four members, BRD2, BRD3, BRD4 and BRDT. Through their extensive molecular functions, they regulate chromatin architecture and transcription. Their deregulation leads to a wide array of diseases, ranging from cancer, viral infections and metabolic diseases to mental disability. BRD4 is the most studied member of the BET family being structurally and functionally unique with an extended C-terminal tail, a defined dimerization motif and intrinsic kinase as well as HAT activities. Despite the development of several pan BET inhibitors as potential therapeutic agents, specific targeting of individual BET proteins remains challenging since most drugs target the structurally conserved bromodomains. There is thus a need to understand the additional roles and mechanisms of individual BET members to develop more effective therapeutics.

The main objective of this Research Topic was to put together a collection of articles to better understand biochemical activities and functions of BET proteins. In all, six articles by experts specializing in different aspects of BET protein biology were published under this Research Topic. These articles are summarized below and document the wide range of biological processes and diseases regulated by BET proteins. We hope that this collection will serve both as a primer to the field and a snapshot of future directions.

Cornelia de Lange Syndrome (CdLS) is a genetic mental disability traditionally linked with the mutations in core (SMC1A, SMC3 and RAD21) and regulatory (NIPBL) subunits of cohesin with up to 70% of cases associated with NIPBL mutations. The sister chromatid cohesion function of cohesin is not affected in CdLS, instead the etiological basis of CdLS has been linked with transcriptional dysregulation. The review by García-Gutiérrez and Mario García-Domínguez discusses the roles of chromatin factors, and their potential mechanisms of action, in CdLS, with an emphasis on BRD4. BRD4 has been recently identified as one of the proteins mutated in CdLS-like phenotypes, a finding that was explained by showing an interaction between the BRD4 and NIPBL. This review discusses the cohesin complex, NIPBL and BET proteins in the context of CdLS. Further, the review emphasizes the partnership between NIPBL and BRD4, supporting the notion that transcriptional dysregulation is the main cause for developmental defects in CdLS.

BRD4 has been shown to regulate the innate immune responses by modulating the expression of cytokines. The research article by Xu et al. extends the role of BRD4 in innate immune responses and identifies additional gene regulatory networks during respiratory syncytial virus (RSV) infection. Using human small airway epithelial cells as a model system, the authors compared RNA profile in cells infected with RSV in the presence or absence of ZL0454, a potent BRD4 bromodomain inhibitor. Authors identify that BRD4 not only dynamically regulates the expression of RSV induced cytokines and extracellular matrix (ECM) genes but also regulates the expression of its own gene and its interactome in response to RSV infection. The finding that the interaction of BRD4 with NF-κB subunit RelA was bromodomain dependent and chromatin accessibility data corelated with ECM gene expression in response to RSV infection further supported their observations. Overall, this article provides new insights on the role of BRD4 in antiviral host response.

The review by Cheung et al. provides a birds-eye view of overlapping and distinct functions of BET proteins in transcription and their association with general biology and diseases. Notably, the mechanisms that regulate BET protein expression, such as regulation of BRD4 transcription by 5-hydroxymethylcytosine, mRNA stability by miRNA and translation by METTL3, are discussed with respect to its effects in various diseases. The regulation of BET protein functions by post-translational modifications and interaction with nucleic acids as well as the many protein-protein interactions with BET proteins are discussed. How these regulatory effects modulate gene transcription and splicing, chromatin remodeling, DNA replication and DNA damage repair are reviewed and discussed. Finally, the article discusses the possible harnessing of context-dependent activator and repressor functions of BET proteins for future development of effective therapeutic agents against cancer and inflammatory diseases.

Carbohydrate mediated induction of metabolic genes is modulated by histone acetylation and the histone acetylation reader BRD4. The review by Mochizuki et al. provides an overview of carbohydrate-responsive metabolic gene regulation through transcription factors and epigenetic memory in promoter-enhancer regions as well as histone acetylation and BRD4 in the gene body. The regulation of transcription initiation and elongation of carbohydrate responsive genes in several different tissues and organs through histone acetylation and BRD4 is reviewed and summarized. The review postulates that metabolic diseases such as diabetes are induced by reducing the expression of BRD4 targeted carbohydrate-responsive metabolic genes in white adipose tissue. On the other hand, inducing the expression of BRD4 targeted metabolic genes in the liver, small intestine and innate leukocytes could have the same effect.

McBride et al. review numerous roles of BRD4 in the infectious cycle of human papillomaviruses. The article introduces human papilloma viruses (HPV) and their association with diseases. It then describes the infectious cycle of HPV with an emphasis on genomic regulation including transcription and DNA replication at various stages. The interesting dual role of BRD4 in viral transcription as an activator at very early stages of infection, and next as a co-repressor during early gene transcription by physically associating with E2, are described in detail. Further, the authors discuss BRD4’s role during different stages of the infectious cycle of HPVs. Importantly, the authors shed light upon how viral genome integration is linked with carcinogenesis and the contribution of BRD4 therein. Finally, authors discuss the potential application of BRD4 small molecule inhibitors as therapeutics for HPV infections and associated cancers.

Liu et al., systematically review the structural and functional characteristics of BET proteins, with a special focus on their role in inflammation and CNS diseases. In addition to a comprehensive review of BET protein structure and BET inhibitors, the authors also provide mechanistic details of different anti-inflammatory pathways such as NF-κB and Nrf2 pathways, and the interplay between BET proteins and these signaling pathways. Further, the authors discuss BET proteins in the pathogenesis and progression of multiple neurological inflammatory pathologies. Finally, they present a future perspective of potentially using genetic and pharmacological approaches to further understand the mechanistic role of BET regulation in these pathologies.

In summary, this Research Topic covers a gamut of observations, perspectives, and ideas from BET protein experts that we hope will generate much interest and discussion in the field.

